# Revolutionizing orofacial pain management: the promising potential of stem cell therapy

**DOI:** 10.3389/fpain.2023.1239633

**Published:** 2023-11-13

**Authors:** Ke Ren, Russel Vickers, Josue Murillo, Nikita B. Ruparel

**Affiliations:** ^1^ Department of Pain and Neural Sciences, University of Maryland, Baltimore, MD, United States; ^2^ Clinical Stem Cells Pty Ltd., Sydney, NSW, Australia; ^3^ Oral Health Center, School of Dentistry, Faculty of Health and Behavioural Sciences, The University of Queensland, Brisbane, QLD, Australia; ^4^ Institute for Glycomics, Griffith University Queensland, Southport, QLD, Australia; ^5^ Department of Endodontics, University of Texas Health Science Center at San Antonio, San Antonio, TX, United States

**Keywords:** orofacial pain, stem cell analgesia, MSC secretome, trigeminal pain, mechanisms of stem cell anti-nociception

## Abstract

Orofacial pain remains a significant health issue in the United States. Pain originating from the orofacial region can be composed of a complex array of unique target tissue that contributes to the varying success of pain management. Long-term use of analgesic drugs includes adverse effects such as physical dependence, gastrointestinal bleeding, and incomplete efficacy. The use of mesenchymal stem cells for their pain relieving properties has garnered increased attention. In addition to the preclinical and clinical results showing stem cell analgesia in non-orofacial pain, studies have also shown promising results for orofacial pain treatment. Here we discuss the outcomes of mesenchymal stem cell treatment for pain and compare the properties of stem cells from different tissues of origin. We also discuss the mechanism underlying these analgesic/anti-nociceptive properties, including the role of immune cells and the endogenous opioid system. Lastly, advancements in the methods and procedures to treat patients experiencing orofacial pain with mesenchymal stem cells are also discussed.

## Introduction

The orofacial region commonly experiences persistent myogenic and neurogenic pain, which poses a significant health concern. Acute orofacial pain is often a response to identifiable triggers, such as tissue damage, pathological conditions, and diseases affecting the mouth, face, and jaw region. On the other hand, chronic orofacial pain defined as pain occurring for more than 15 days per month and lasting for more than 4 h daily for at least the last 3 months (1), can endure even in the absence of tissue damage and may persist after the resolution of a pathological condition. This key distinction highlights that chronic pain mechanisms are more complex, involving altered neural processing and sensitization, leading to a prolonged and persistent pain experience. Managing chronic orofacial pain may require a comprehensive and multidisciplinary approach to address the underlying mechanisms contributing to its persistence. Chronic orofacial pain is prevalent in the United States and impacts approximately 20% of the population (2). Moreover, treating chronic orofacial pain patients costs over $32 billion each year and has contributed to the opioid epidemic for decades. Despite this, the discovery of most novel non-opioid treatment options for the management of orofacial pain has been poor. TMJD (temporomandibular joint disorders) is the most common orofacial pain condition, affecting musculoskeletal and joint tissues, reducing quality of life. Unfortunately, effective treatment options for this condition are lacking, especially in light of the current opioid epidemic, which necessitates alternative pain management strategies. In recent years, cell-based pain management has shown promising results. Mesenchymal stem cells (MSCs) have received attention due to their ability to differentiate into osteoblasts and chondrocytes (3, 4), and they have been used to repair damaged joint tissues while providing pain relief. In an earlier veterinary clinical study, injection of adipose MSCs attenuated pain in dogs suffering from hip osteoarthritis (OA) (5). Many subsequent studies have shown the beneficial effect of MSCs in arthritic joint pain (6, 7).

**Figure 1:**
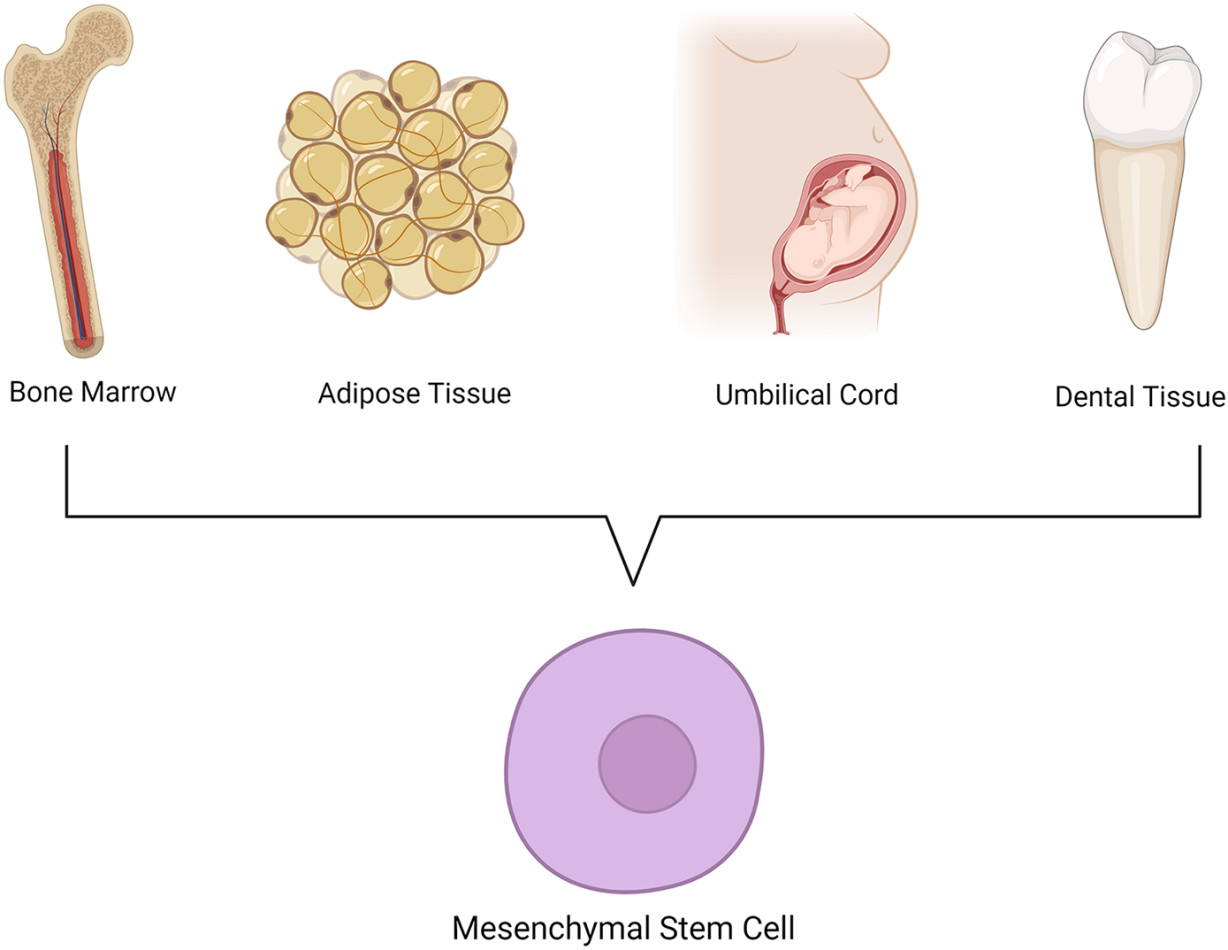
Common tissue sources of stem cells.

MSCs are a population of stromal cells capable of self-renewal and differentiation into different cell types, including osteoblasts, chondrocytes, and adipocytes. They are critical for the regeneration of new tissue in order to maintain homeostasis and functional health in humans. They are found in the tissues of all species throughout the plant and animal kingdoms. MSCs have been isolated from various tissues, including bone marrow, adipose tissue, umbilical cord, and dental tissue (Figure 1). At first, interest in using mesenchymal stem cells was due to their potential use in wound healing and regeneration. However, over the past decade, advancements have also shown that MSCs produce robust pain relief in a variety of conditions ranging from neuropathic pain in mice to osteoarthritis and migraines in patients. The immunosuppressive and immunomodulatory properties of stem cells have been shown to contribute to this pain relief (8, 9). Additionally, the secretome of stem cells are also sufficient to produce these effects, suggesting that paracrine release of cytokines and other factors are necessary. Interestingly, most, if not all, mesenchymal stem cells have been shown to have these pain-reliving properties, yet impactful differences and advantages/disadvantages can still be observed. In this review, we will cover three main topics related to MSCs and their potential for pain management:

(1) A comprehensive analysis of different sources of MSCs and their preclinical outcomes in pain management.
(2) Mechanisms underlying MSC-mediated anti-nociception.
(3) An examination of protocols and procedures for the use of MSCs in clinical applications of orofacial pain.

## Mesenchymal stem cell types: outcomes of preclinical outcomes

### Bone marrow stem cells (BMSCs)

BMSCs are a population of stromal cells isolated from the bone marrow space. These cells are capable of differentiating into muscle, bone, and epithelial linages (3, 10). BMSC are the most widely studied source of stem cells for the treatment of pain in animal and clinical research, mainly due to the accessibility of these cells and their abundance. Reversal of a variety of pain models by BMSC administration has been demonstrated in both the dorsal root and trigeminal ganglia. Intraganglionic and systemic administration of rat BMSCs reverses mechanical and thermal hypersensitivity induced by chronic constriction injury (CCI) of the sciatic nerve (11, 12). Similar effects are also observed is rats with diabetic neuropathy (13), spared nerve injury (14), and osteoarthritis (15). For the orofacial region, BMSCs reverse mechanical and thermal nociception induced by tendon ligation of the master muscle and CCI of the infraorbital nerve in rats (8, 16, 17). According to Guo and colleagues' study in 2011, rats showed reduced mechanical hypersensitivity after receiving intravenous administration of BMSCs diluted in PBS at 7 days after ligation injury of the masseter muscle tendon (TL). BMSCs were derived from adult Sprague-Dawley rats grown in the same plate without further passage and the cells were harvested at 7 days after initial plating. Notably, one dose of BMSCs at 1.5 M cells produced pain relief that lasted for months. Similar long-lasting antihyperalgesic effects of BMSCs existed in animals with chronic constriction injury of the infraorbital nerve (CCI-ION), a model of orofacial neuropathic pain (16). It is worth noting that the administration of BMSCs did not have an impact on the rat's performance on the rotarod test, indicating that the observed antihyperalgesic effect, as evaluated by reflex measurement, was not due to locomotor impairment. In addition to reflex measures of mechanical nociception, the pain-related aversive behavior assessed by a conditioned place avoidance test (18) was reduced after the treatment with BMSCs (17, 19). The reduced voluntary biting behavior, a measure of functional orofacial nociception, was also seen to be improved in mice with the injury of the masseter muscle tendon after infusion of BMSCs (19). The face grooming behavior after CCI-ION improved by injection of BMSCs into the trigeminal ganglion (20). In *ex vivo* trigeminal medullary slices dissected from CCI-ION mice, the BMSC treatment significantly reduced the amplitude and frequency of spontaneous excitatory postsynaptic currents (sEPSCs) in lamina II neurons when compared to that from culture medium-treated mice (17). The GluN (N-methyl-D-aspartate) receptor-mediated evoked EPSCs (Excitatory PostSynaptic Currents) were increased in CCI-ION mice but inhibited by the BMSC treatment (17). These observations provide convergent evidence of BMSC-produced behavioral pain relief associated with orofacial injury and indicate suppression of trigeminal neuronal hyperexcitability and primary nociceptive afferent input at the trigeminal nucleus by BMSCs. It is noted that MSCs can be primed or modified to improve their therapeutic effect. IL-1β-pretreated MSCs have shown improved antihyperalgesia (21, 22). TLR3-primed MScs are immunosuppressive, in contrast to TLR4-primed MSCs that are pro-inflammatory (23). BMSCs with inserted human preproenkephalin gene produced a strong analgesic effect in a rat neuropathic pain model (24).

Due to the extensive literature associated with BMSCs and pain, several cell-based features emerge for the use of BMSCs and may have clinical strong implications: (1.) The pain-relieving effect of MSCs in humans is directly related to the number of cells that are transfused, with a larger number of cells producing greater pain relief (6). In the masseter muscle TL rat model, it was found that injecting 1.5 million BMSCs produced anti-hyperalgesia, while the injection of 1.5 thousand cells was ineffective (16). Moreover, in rats, it was observed that direct injection of BMSCs into the injured site required about 0.4 million cells for anti-hyperalgesia (16). (2.) It has been noted that the antihyperalgesic property of BMSCs is lost after high passages in culture. Primary BMSCs, but not BMSCs after 20 passages, attenuated hyperalgesia in the masseter muscle TL rats (16). Prolonged culturing of BMSCs can result in a loss of their ability to differentiate into multiple lineages and effectively home (25, 26). Furthermore, studies have demonstrated that repeated passaging of MSCs in culture, particularly beyond 15 passages, can induce significant phenotypic changes (27). Specifically, a culture with two-passage human BMSCs expresses a distinct set of chemokine receptors that are not found in 12–16 passage cells (28). Thus, altered genotype during repeated culturing may render high passage BMSCs ineffective against pain hypersensitivity. Nevertheless, high-passage BMSCs can be used as an appropriate control in studying the cellular and molecular mechanisms of the therapeutic effect of BMSCs. Collectively, the number, as well as the stage of BMSCs, are crucial for their use as potential anti-hyperalgesic cell types.

Studies utilizing human-derived cells in preclinical and clinical studies have also demonstrated anti-nociceptive properties (17, 29, 30). Specifically, spinal nerve ligation-induced mechanical and thermal nociceptive behavior is reversed with human BMSCs (17). Furthermore, BMSC-induced analgesia has been demonstrated in patients with osteoarthritis (29, 30). Interestingly, it seems that BMSCs may be better suited for treatment of conditions involving bones and joints. One study comparing the properties of BMSC to other stem cells found that they have a higher differentiation rate into osteogenic linage (31). Furthermore, BMSCs have a higher capacity for mineralization *in vitro* when compared to adipose-derived and dental pulp stem cells (DPSCs) (32). The aforementioned indicates that stem cells sourced from diverse tissues and developmental origins may possess distinctive characteristics that are appropriate for certain disease conditions, a subject that will be elaborated on in the subsequent sections. Nonetheless, despite the encouraging signals regarding the application of BMSCs, they are not without limitations. Specifically, the proliferative capacity of BMSCs is less than satisfactory when compared to adipose-derived and umbilical cord stem cells (UCSCs), with BMSCs exhibiting the lowest proliferative capacity (33). Additionally, the isolation of BMSCs from humans involves an invasive procedure that can lead to adverse effects and complications. Patients report pain at the site of donation, severe vomiting and headaches, and experience restricted mobility of the limb for up to 28 days after BMSC isolation (34). Nevertheless, additional information regarding the complications arising from BMSC isolation is required since the existing studies have certain limitations in terms of sample size and conclusions drawn (34). Notwithstanding the need for more comprehensive research, it is worth noting that the application of BMSCs has provided compelling evidence for stem cell-based analgesia.

### Adipose-derived stem cells (ADSCs)

Research involving ADSCs has been on a steady rise. Issues with the isolation of BMSC from patients discussed above, along with low stem cell count during collection (3), have led some to find an alternative. ADSCs are found in white adipose tissue deposits located around the thighs and abdomen of patients and can be harvested from liposuction procedures that bring minimal risks. This adipose tissue that would otherwise be discarded has been shown to yield higher amounts of stem cells (35) and faster proliferation rates (33, 36) when compared to bone marrow. Additionally, ADSCs have been shown to differentiate into an adipocyte lineage at higher rates than BMSCs (32), suggesting that these cells may be better suited for wound healing. Additionally, properties similar to BMSCs have also been demonstrated in the treatment of pain in animal and human studies (9, 37–41). While research on ADSCs for orofacial pain is not as common as for BMSCs, a clinical study presents promising results in patients with migraines (38). Moreover, studies on tissue innervated by dorsal root ganglia (DRG) neruons reveal comparable anti-nociceptive characteristics. A single subcutaneous hindpaw injection of ADSCs was shown to restore nociceptive behaviors back to baseline in a burn injury model of pain (37). Anti-nociception was first detected three weeks after injection and lasted for the remainder of the testing period, two weeks (37). This injection decreased COX-2 levels in both the site of injury (hindpaw) and the spinal cord (37). ADSCs and their secretome can also reduce nociception in mice with diabetic neuropathy (9). Systemic injection of ADSC reverses mechanical hypersensitivity as soon as three hours post-injection, with a maximal effect two weeks after injection. Importantly, this reduction was long-lasting as mechanical thresholds were increased for up to 12 weeks post-injection (9). A decrease in inflammatory cytokines IL-6 and TNF*α* was observed in the siatic nerve, DRG, and spinal cord 2 weeks following systemic injection of ADSCs and it's secretome (9). Furthermore, the anti-inflammatory cytokine IL-10 was increased, while levels of pro-inflammatory IL-1β were decreased in the spinal cord up to six weeks after ADSC injection and 4 weeks after ADSC were still found in tissue (9). This long lasting effect ADSC could be mediated by priming of the immune system to restore pro- and anti-inflammatory immune cell and cytokine balance, a topic discussed later in this review. Additionally, systemic, intraplantar, and intra-articular injection of ADSCs-derived conditioned media inhibits thermal and mechanical hypersensitivity in a rodent model of the OA (40). It is important to note that variability in the onset of anti-nociception after ADSC injections can vary between studies and may depend of factors such as source and isolation of the stem cell population, concertation used, and route of administration. Clinical studies utilizing ADSCs have also shown success. Intra-articular injection of ADSCs led to pain improvement lasting up to 6 months in patients diagnosed with OA (41). Studies investigating the use of these stem cells for the treatment of migraines and lower back pain have also displayed promising results (38, 39). Collectively, these data provide support for the use of ADSCs as an anti-nociceptive cell population and an alternative to BMSCs.

### Umbilical cord stem cells (USCSs)

The use of UCSCs as an alternative source of stem cells has been rapidly gaining popularity. These cells can be safely obtained through venous puncture of the umbilical cord, with minimal risk to both the patients and the cord tissue following birth (42). In the case of the latter, umbilical cord tissue would otherwise be discarded, leaving many eager to collect this tissue for stem cell isolation. With more than 3.6 million births reported in the United States in 2021 (43), UCSCs are seen as a potentially abundant source for research. The advantages of using UCSCs include faster proliferation rates than ADSCs and BMSCs (33). This is mainly attributed to these cells being more primitive than stem cells taken from adults. However, there are drawbacks to the isolation of UCSCs. Specifically, isolation from cord blood has been shown to yield low UCSC count due to the limited number of blood available to collect (44). Studies have shown that UCSCs are only isolated from 29%–63% of cord blood collected from patients (33, 45, 46). As MSC can typically be isolated from 100% of bone marrow and adipose tissue samples taken from donors (33, 46). Additionally, there is also variability between UCSCs isolated from donors. One study found donor dependent variability in UCSCs angiogenic ability (47), while another found variability in proliferation rates and immunomodulation from cells isolated from 32 patients (48). Overall, both the low cell yields and donor variability from umbilical cords poses an obstacle for wide adoption of UCSC (33, 46, 49). Research into the analgesic properties of UCSCs in orofacial pain models is also sparse, but their effect on neuropathic pain induced by spinal cord injury has been well characterized. Rats having undergone spinal nerve ligation show increased mechanical and thermal thresholds after intrathecal injection with UCSCs (50, 51). Similar results were also observed after intrathecal injection of UCSCs in mice with spinal cord injury (SCI). In this study, UCSCs exhibited higher survival rates in the spinal cord compared to BMSCs. Additionally, neurons harvested from UCSC-treated mice displayed decreased stimulation responses and windup when compared to mice treated with BMSCs (52). Furthermore, another study comparing the effects of ADSCs and UCSCs showed that both are able to reverse mechanical nociception in rats with sciatic nerve ligation. However, rats treated with UCSCs showed almost full reversal of demyelination in sciatic nerve fibers, when compared to vehicle and ADSCs treated mice (53), highlighting a possibility that stem cells from different tissues may all induce analgesia but through unique mechanisms.

### Dental stem cells (DSCs)

DSCs are harvested from a variety of tissues in the oral region. The predominant DSCs include dental pulp stem cells, periodontal ligament stem cells, stem cells of the apical papilla, and stem cells from human exfoliated deciduous teeth. Similar to ADSCs and UCSCs, dental stem cells are seen as an attractive alternative as these cells are harvested from tissues that would otherwise be discarded. Additionally, dental stem cells have been shown to have faster proliferative rates when compared to BMSC (31). DSCs have mainly been studied for their potential role in tooth regeneration. For example, dental pulp stem cells (DPSCs) and stem cells from human exfoliated teeth (SHED) have been extensively looked at for their potential role in dentin reformation and dental pulp regeneration (54, 55). One distinctive feature of dental stem cells is their origin from the neural crest, in contrast to BMSCs and ADSCs, which originate from the mesoderm. This neural crest origin categorizes dental stem cells as ectomesenchyme, derived from neural crest tissue that develops into the head and neck. Due to their neural crest origin, dental stem cells may possess superior neuro-protective and neuro-regenerative properties compared to the aforementioned stem cells (56). DPSCs have been shown to secret neurotrophic factors and promote survival and growth of trigeminal ganglionic neurons *in vitro* and rescue motor neurons *in vivo* (57, 58). DPSCs also express higher levels of neural stem cell markers and secrete higher amounts of wound healing cytokines, such as transforming growth factor-beta, vascular endothelial growth factor, and nerve growth factor, than BMSCs (58, 59). Additionally, transplantation of stem cells of the apical papilla (SCAP) improved motor function to baseline levels in rats with spinal cord injury (60). Furthermore, in a model of spinal cord injury, rats transplanted with DPSCs or SHEDs, but not BMSCs, regained motor function within 5 weeks after transplantation (61). A significant effect was also observed *in vitro* as conditioned media from DPSCs and SHEDs induced marked neurite extension compared to that from BMSCs (61). The ability of DSCs to promote neurite growth through the paracrine release of neurotropic factors such as brain-derived and glial cell line-derived neurotrophic factors plays a crucial role in mediating the observed superior neuro-protective and neuro-regenerative properties (62, 63). Despite the abundance of research demonstrating the neuroprotective effects of DSCs, their properties for pain relief remain limited. One study has demonstrated that a single injection of DPSCs can reverse hypersensitivity in a mouse model of diabetic neuropathy (64). Additionally, conditioned media from SHEDs was able to partially reverse mechanical nociception in mice having undergone sciatic nerve ligation (65). Furthermore, neuropathic pain in the orofacial region of rats was seen to reduce by SHEDs (66, 67) and that intra-articular injection of DPSCs reduced cartilage matrix degradation, improved bone regeneration, and attenuated hyperalgesia in complete Freund's adjuvant (CFA)/monosodium iodoacetate-induced TMJ arthritis in rats (68). Further investigation is still required to comprehensively determine the pain-relieving characteristics of DSCs and how they compare to stem cells obtained from other sources. Moreover, mechanisms that mediate such effects including the possible opposing effects of neurotrophins such as NGF, BDNF and GDNF, that are known pro-nociceptive mediators (69, 70), particularly in the context of neuropathic pain are warranted.

### MSC secretome

Although MSCs have long been studied for cell based applications, a growing body of evidence suggests that these effects are largely due to paracrine action, rather than MSC differentiation (71, 72). The paracrine release of all the factors from MSC into the extracellular space including proteins, nucleic acids, lipids, and extracellular vesicles is defined as the MSC secretome (73). The use of the secretome is seen as a promising alternative for treatment in patients. Currently, there is concern over the use of MSC therapeutics due to the potential adverse effects, such as administration site reactions, unwanted cell homing and differentiation, and potential tumor formation (74). The use of the secretome through conditioned media or extracellular vesicles eases some of these concerns. Additionally, the use of the secretome allows the evaluation of potential doses and potency similar to conventional drug development. Importantly, the secretome produces similar anti-nociceptive effects in pre-clinical studies when compared to using cells. Systemic injection of conditioned media sourced from ADSC reverses nociception in a model of diabetic neuropathy similar to the use of cells (9). Additionally, the conditioned media produced similar immunomodulatory effects as the ADSC, suggesting that their effects may be attributed to their secretome. Another study demonstrates that ADSC-derived conditioned media can decrease the expression of proinflammatory cytokines and inhibit thermal and mechanical hypersensitivity in a model of OA (40). Furthermore, a single injection of both BMSC- and SHED-derived conditioned media inhibit mechanical nociception in mice having undergone sciatic nerve ligation (65, 75). Local injection of ADSC-derived conditioned media into the knee joint or muscle of patients with musculoskeletal pain significantly improved patient pain scores 1 to 4 weeks after injection with no reported adverse effects (76). Manipulation of MSC through a variety of methods is also seen as a way to increase the effectiveness of MSC secretome treatments (73, 77). Pretreatment or manipulation of MSC with pro-inflammatory factors, hypoxic conditions, and serum-free media has shown promising results. Incubation of BMSC with TNFα and IL-1β increased the release of anti-inflammatory factors into conditioned media when compared to untreated cells (78). TNFα pre-treatment has also been shown to increase the angiogenic activity of ADSC *in vitro* and *in vivo* through the release of IL-6 and IL-8 (79). Additionally, the use of conditioned media from ADSC in hypoxic conditions increased the expression of anti-inflammatory mediators and promoted liver regeneration in mice (80). Only a handful of studies have investigated the use of manipulated MSC for pain. Conditioned media collected from serum starved ADSC is effective in inhibiting nociception induced by models of diabetic neuropathy and OA (9, 40). Despite this promising data, more research is needed to fully understand the effects manipulation of MSC may have on pain conditions. Additionally, there are drawbacks to the use of the MSC secretome. The standardization of secretome collection protocols (cell concentration used, volume, incubation time, cell passage #) is needed to ensure reproducibility across groups, and through analysis of factors released by MSC into collected conditioned media or isolated extracellular vesicles needs to be conducted (81).

## Mechanistic insights into mesenchymal stem cell-induced analgesia

### The engagement of the endogenous opioids

An aforementioned study by Guo et al. demonstrating BMSC-induced decrease in orofacial nociception was reversed by opioid receptor antagonists (8, 16). When naloxone was administered in rats receiving BMSC treatment after a TL of the masseter muscle, the BMSC-produced antihyperalgesia was partially reversed. Naloxone is an inverse agonist that may block the constitutive activity of opioid receptors and lead to increased pain (82). However, the reversal of antihyperalgesia by naloxone is unlikely due to its inverse agonist activity since a neutral opioid receptor antagonist 6-β-naltrexol also induced pain in BMSC-treated rats (8). Naloxone also induced the hyperexcitability of trigeminal neurons recorded from an *ex vivo* trigeminal slice mouse preparation (17). The effect of naloxone on neuronal activity is consistent with behavioral measures, suggesting the involvement of endogenous opioids in BMSC's antihyperalgesia.

The endogenous opioid system exists at multiple levels of the pain modulatory pathways including the cortex, hypothalamus, and brainstem. The rostral ventromedial medulla (RVM) mediates descending input from midbrain pariaqueductal gray and has been established as a key structure in descending pain modulation under both normal and under conditions of injury he endogenous opioid system exists at multiple levels of the pain modulatory pathways including the cortex, hypothalamus, and brainstem. The rostral ventromedial medulla (RVM) mediates descending input from midbrain pariaqueductal gray and has been established as a key structure in descending pain modulation under both normal and under conditions of injury (83, 84). Guo et al. (8) demonstrated that the expression of *μ* (MOR), but not *δ* (DOR) and *κ* opioid receptors (KOR) were upregulated in the RVM after BMSC treatment in masseter muscle TL and CCI-ION rats. The treatment with 20-passage BMSCs did not upregulate MORs, consistent with their ineffectiveness against behavioral hyperalgesia. Most recently, Fernandes et al. (85) demonstrated that the secretome, or conditioned medium of cultured human BMSCs induced a 4-fold increase in MOR in cerebral organoids (a resemblance of the 3D-brain structure generated from human pluripotent stem cells), while the expression of DOR and KOR was not affected. Consistently, specific downregulation of MOR in the RVM by RNA interference led to the reoccurrence of hyperalgesia in BMSC-treated rats (16). The descending pain modulatory pathway can be activated by various modalities, including brain stimulation, stress, vagal afferent input, and acupuncture (86). Electroacupuncture also activates RVM MOR, but not KOR, in producing anti-hyperalgesia (87). Collectively, it is conceivable that MSCs may also potentially be an alternative class of cells that are potent activators of endogenous pain inhibition via the upregulation of MOR in the RVM.

Repeated activation of endogenous opioids can lead to the development of opioid tolerance (88). However, BMSC-induced long-term antihyperalgesia does not seem to result in tolerance, and instead involves the inhibition of the expression of the N-methyl-D-aspartate (NMDA) receptor 2A subunit GluN 2A receptor, which helps protect against glutamate excitotoxicity (89). This is important, as the GluN receptor is known to play a role in the development of opiate tolerance (90). Additionally, the GluN2A subunit of the GluN receptor in RVM is important for descending pain facilitation (91), and its tyrosine phosphorylation in the RVM was found to be suppressed at 8 weeks after BMSC treatment (17). Protein kinase C (PKC)*γ* activity related to GluN receptor activation is critical in opioid tolerance (92), and PKC*γ* immunoreactivity in the RVM was also decreased at 8 weeks after BMSC treatment (17). In summary, the long-term antihyperalgesic effects of BMSCs involve an effect on GluN receptors, which allows for a desirable analgesic profile, specifically promoting opioid analgesia while suppressing the development of opioid tolerance.

Overall, the release of endogenous opioids appears to be one mechanism mediating the antinociceptive effects of MSCs, leading to antihyperalgesia. This effect is particularly effective due to the multiple sites of action of the endogenous opioid system, while avoiding the negative side effects of opioid tolerance.

### Role of immune cells

Mechanisms other than a direct effect or engraftment of BMSCs underlie their long-lasting pain-relieving effect. This is concluded due to several lines of evidence: (1) Most intravenously infused MSCs are trapped in the lungs e.g., traced with BMSCs from GFP transgenic rats (7), or Qtracker-labeled cells (93). Even a direct infusion of BMSCs into the external carotid artery that can bypass the first-pass effect of the lung only leads to transient recruitment of the cells to the brain for about 24 h (94); (2) Studies utilizing BMSCs from GFP transgenic rats have demonstrated that GFP-labeled BMSCs were clustered in the lungs within one day after systemic infusion, and very few GFP-positive cells remained there at 7 days after infusion (8); (3) The cells trapped in the lungs disappeared with a half-life of ≈24 h, and many of them would undergo apoptosis (95); (4) At four days, only about 0.01% of the systemically infused cells were recruited to the spleen, liver, kidney, heart, pancreas, and brain in the mouse (95); (5) In rats, a very small number of cells (0.0005%) could reach the injured brain site (96, 97); (6) After a para-ganglionic injection of BMSCs, no labeled BMSCs were localized in the trigeminal ganglion at 24 days post-treatment (67), and (7) MSCs appear to survive better after intrathecal administration. About 50% of intrathecally administered MSCs were distributed to the dorsal root ganglion and survived there for 1–2 weeks (98).

Current literature supports a hypothesis that the pain-relieving effect of transplanted MSCs is a result of their interactions with host immune cells. After systemic administration, MSCs are exposed to circulating immune cells (99). Even cells embolized in the lungs can interact with host immune cells and secrete anti-inflammatory mediators (95, 100). It is well established that the interactions between the immune system and pain pathways contribute significantly to the development of chronic pain (101). Thus, it was speculated that infused MSCs may first engage in a talk with host immune cells, leading to the release of immune mediators and activation of the anti-inflammatory signaling cascades leading to activation of the endogenous analgesic system for the observed antihyperalgesic effects of MSCs (discussed above).

Numerous studies have investigated the role of monocytes/macrophages in BMSC-induced antihyperalgesia. It has been demonstrated that the monocyte/macrophage population mediates the anti-inflammatory effect produced by BMSCs (100). Brack et al. (102) reported that partial depletion of monocytes/macrophages reduced peripheral opioid-induced analgesia. Additionally, macrophages have been shown to produce an anti-inflammatory and pro-resolving mediator, maresin 1, which relieves neuropathic pain in mice (103). Macrophages were also seen to be recruited to the cerebrospinal fluid by IL-10 encapsulating plasmid DNA-containing microparticles, which correlated with the relief of experimental neuropathic pain for more than 74 days (104). Furthermore, there is direct evidence for the involvement of monocytes/macrophages in BMSC-produced orofacial hyperalgesia (8). They observed that pre- and post-treatment with liposome-encapsulated clodronate led to partial depletion of macrophages in the spleen and circulation, as well as attenuation of BMSC-produced antihyperalgesia in the masseter muscle TL rat model. Additionally, upregulated MOR mRNAs in the RVM were reduced after monocyte/macrophage depletion and that hyperalgesia was also reduced after intra-RVM injection of peripheral blood mononuclear cells (PBMCs) isolated from TL rats receiving BMSC treatment. These studies collectively provide strong support for the involvement of the monocyte/macrophage population in the BMSC's antihyperalgesic effect.

**Figure 2:**
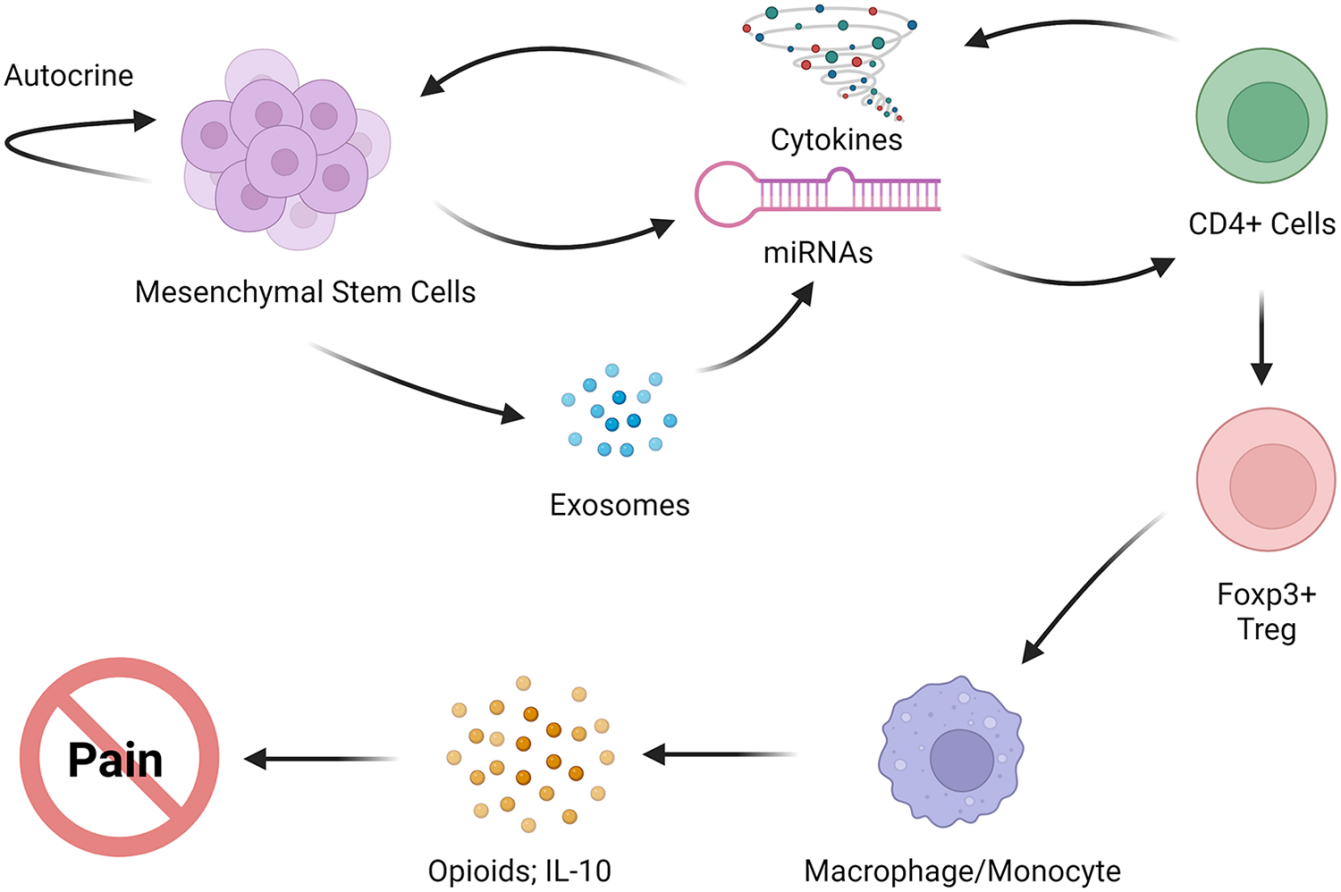
Proposed immune interactions of MSCs with host immune cells that lead to anti-inflammatory phenotype, the release of endogenous opioids, and pain relief.

The involvement of BMSCs in modulating macrophages is observed to have a particular impact in promoting the anti-inflammatory M2 macrophage phenotype (105–107). The reduction in BMSC-induced neuropathic pain is associated with an increased expression of CD206, a marker of M2 macrophage in the spinal cord in mice (14). Additionally, an increase in transcription of M2 markers, namely, *CD206*, *CD163,* and *Irf4* in PBMSCs derived from BMSC-treated TL rats has also been observed (8). The *Irf4* transcription factor has been shown to control M2 macrophage polarization (108). Resident microglia may also be modulated to express M2 phenotype after BMSC treatment (109). In the rat model of persistent pain induced by TL of the masseter muscle, transplantation of BMSCs upregulated CD206 associated with microglia in the RVM (110). These observations point to the role of alternatively activated macrophages and brain microglia in the pain-relieving effect of MSCs. The regulatory T cells (Tregs) are derived from CD4^+^ T cells and are key to immune homeostasis. The CD4^+^CD25^+^Foxp3^+^ Tregs have been shown to induce alternative activation of monocytes/macrophages (111). Deletion of Tregs led to increased pain after nerve injury (112). Compared to naïve mice, the frequency of CD4^+^CD25^+^Foxp3^+^Tregs among the CD4^+^ population was decreased in mice with CCI-ION. However, their frequency was significantly increased to above baseline levels 8 weeks after BMCS treatment in the nerve-injured mice (113). These findings support a scenario in that BMSC-secreted mediators induce Tregs, followed by subsequent activation of the anti-inflammatory M2 phenotype, leading to the observed long-lasting pain relief (Figure 2).

### Role of immune mediators

The anti-hyperalgesic effects of MSCs have been associated with the regulation of inflammatory mediators, which contributes to their anti-nociceptive phenotype. Numerous lines of evidence support this assertion:

MSCs have been shown to promote an anti-inflammatory response by downregulating pro-inflammatory IL-1β and upregulating anti-inflammatory IL-10 (14, 114). Additionally, the inhibitor of cytokine transcription pathways, suppressor of cytokine signaling 3 protein (SOCS3), was found to be increased in the RVM after treatment with BMSCs, consistent with the inhibition of IL-1β (110). In experimental TMJ arthritis, local intra-TMJ injection of DPSCs improved hyperalgesia and reduced inflammatory TNF and IFN-γ expression through inhibition of the signal transducer and activator of transcription 1 (STAT1) signaling (68). The pain-relieving effect of BMSCs was found to be reduced when the expression of anti-inflammatory TSG-6 (TNF-stimulated gene 6 protein) was inhibited (115). Moreover, MSCs from adipose tissues of osteoarthritis patients have been shown to have a proinflammatory cytokine profile involving STAT3. However, blocking STAT3 expression resulted in lower levels of proinflammatory cytokines and higher levels of anti-inflammatory cytokines, making them more therapeutically effective (116).

Differential cytokine/chemokine profiles have been observed in primary and 20-passage BMSCs (8). While Ccl4, a monocyte chemoattractant, was highly expressed in primary BMSCs, it was absent in 20-passage BMSCs. Knockdown of Ccl4 from BMSCs led to a significant reduction in their antihyperalgesic effect (8), suggestive of its key role in this process. Additionally, CCR2, which promotes monocyte chemotaxis, was highly expressed in primary BMSCs (8). Pretreatment of BMSCs with a CCR2 antagonist reduced their antihyperalgesia and resulted in reduced MOR expression in the RVM (8). TGF-β, an anti-inflammatory cytokine, was also shown to be crucial for the pain-attenuating effect of BMSCs and was expressed at higher levels in primary BMSCs (8). These findings suggest that MSCs produce their antihyperalgesic effect by secreting immune mediators and interacting with host immune cells.

MSCs may pass immunoregulatory signals to circulating immune cells such as monocytes to produce and maintain their therapeutic effect. CXCL1 (CINC1) chemokine belongs to the CXC chemokine family and signals through its receptor CXCR2. Interestingly, the *Cxcl1* gene showed significantly higher levels of expression in PBMCs after treatment with primary BMSCs compared to those treated with 20-passage BMSCs (8). An increase in CXCL1 proteins in serum and cerebrospinal fluid was identified after the BMSC treatment. Selective *Cxcl1* gene upregulation in PBMCs and the appearance of CXCL1 in the serum and cerebrospinal fluid suggest that CXCL1 may be released after interactions between BMSCs and PBMCs and reach the brain to relay the effect of BMSCs. This hypothesis is supported by the finding that injection of serum from BMSC-treated animals into the RVM upregulated MOR mRNAs and produced antihyperalgesia that was partially blocked by the treatment with anti-CXCL1 antibodies (8). CXCL1 is involved in the regulation and release of opioids from immune cells via its receptor CXCR2 (117). CXCR2 is localized in the CNS (118, 119). CXCR2 immunostaining was found to colocalize with MOR in RVM neurons (8). The enhanced CXCL1-CXCR2 signaling in the brain after BMSC infusion contributed to BMSC-produced descending pain inhibition. After transplantation of BMSCs in the masseter muscle TL rodent model, CXCR2 was upregulated in the RVM, and BMSC-produced antihyperalgesia was attenuated by intra-RVM injection of the CXCR2 antagonists SB225002 and NVP CXCR2 20, down-regulation of *Cxcr2* by RNAi, and knockout of CXCR2 in mice, associated with decreased MOR gene expression (8). These observations indicate that BMSC-immune cell interactions engage pain modulatory circuitry via monocyte-derived CXCL1, leading to lasting pain attenuation in animals with orofacial tissue injury.

It is noteworthy that most chemokines and their receptors mediate chemotaxis and are proinflammatory responders. Studies have shown that the CXCL1-CXCR2 signaling is proinflammatory and pronociceptive (119–123). However, the contribution of CXCL1-CXCR2 signaling in BMSC-produced antihyperalgesia supports the view that the same chemical mediators may play dual roles in pain and analgesia. Similarly, hyperactivity of the glia not only promotes pain hypersensitivity but also anti-nociception, depending on the condition (124, 125). In the experimental autoimmune encephalomyelitis mouse model, overexpression of CXCL1 is anti-inflammatory and reduces autoimmune demyelination (126). Furthermore, activation of CXCR2 on granulocytes induces a signaling cascade that leads to the release of endogenous opioid peptides and analgesia (117). Moreover, the NF-*κ*B protein complex, a transcription factor that controls the transcription of genes involved in immunity, also has dual roles in immune regulation (127). The NF-κB activation induces proinflammatory cytokines and promotes pain hypersensitivity (128). However, the NF-*κ*B signaling pathway in the descending circuitry is involved in BMSC-produced antihyperalgesia (129). NF-κB activation upregulates CXCL1-CXCR2 signaling (130), contributes to MSC-induced neuroprotection (109), and mediates TNF-induced MOR expression *in vitro* (131). Multiple lines of evidence have indicated a facilitatory role of proinflammatory mediators in the anti-inflammatory effect of MSCs. TNF promotes anti-inflammatory IL-1ra release from MSCs (132). CCL4 plays a role in the development of neuropathic pain (133), but is required for BMSC's antihyperalgesia in the orofacial pain model (8). It was found that pro-inflammatory priming promotes the pain-relieving effect of MSCs. BMSCs produced greater inhibition of neuropathic pain in animals after priming BMSCs with proinflammatory cytokine IL-1β (134). IFN-γ is required for the inhibition of B cells by MSCs (135). A recent report indicates that the use of NSAIDs reduces the therapeutic efficacy of MSCs in a rodent OA model (136), suggesting a role of inflammatory response in MSC's beneficial effect. Thus, MSCs-produced pain relief is underlain by sophisticated reciprocal interactions between implanted cells and immune cells and the involvement of both pro- and anti-inflammatory mediators and their receptors. It is unclear whether MSC would be a pain-reliever in immunocompromised patients including those infected with HIV. The pain-relieving effect of MSCs was associated with expanded-CD4 regulatory T cells (22), which may be indictive of a beneficial effect for HIV patients. However, the property of MSCs may be altered after HIV exposure and they may lose their immunosuppressive effect (137).

### The role of MSC-derived exosomes

Exosomes are nano-sized small extracellular vesicles (≈40–150 nm) derived from the endosomal compartment after the fusion of multivesicular bodies with the cell membrane and extracellular release of intraluminal vesicles. Studies indicate that the therapeutic effect of MSCs can be mimicked by concentrated conditioned medium from MSCs (75, 138). Ogasawara et al. (139) showed that a conditioned medium from SHED promoted regeneration and tissue repair in experimental TMJ osteoarthritis in mice. The therapeutic effect of MSC-produced conditioned medium is attributed to MSC-secreted exosomes (140). In a severe graft-vs.-host disease case that did not respond to immunosuppressive interventions, Kordelas et al. (141) showed that MSC-derived exosomes improved the graft-vs.-host disease symptoms shortly after the administration of MSC-derived exosomes.

MSC-derived exosomes are promising surrogates of MSC-based pain relief (110, 142, 143). Experimental neuropathic pain was attenuated by infusion of SHED and conditioned medium, or MSC-derived exosomes (65, 144, 145). MSC-derived exosomes attenuated persistent pain in animal models of osteoarthritis (146, 147). In a TMJ-OA rat model, MSC-derived exosomes attenuated inflammation and pain and promoted joint repair (148).

The pain-relieving effect of MSC-derived exosomes is underlain by mechanisms similar to their parent MSCs (110). Kordelas et al. (141) demonstrated that the exosome preparations contained high quantities of the anti-inﬂammatory molecules, IL-10 and transforming growth factor-β. Conditioned medium from SHED attenuated neuropathic pain involving induced anti-inflammatory M2 macrophages (65), and suppressed activation of microglia and astrocytes (145). In the monoiodoacetate-induced TMJ-OA rat model, BMSC-derived microvesicles improved condylar TMJ histology while reduced IL-1b, TNF, NF-kB, MMP-13, and MMP-3 levels (149). Immunosuppression by MSC-derived extracellular vesicles was enhanced by priming MSCs with inflammatory cytokines (150), an effect similar to that observed on MSCs. MSC exosomes also suppress hyperactivity of glia, as shown by reduced glia marker GFAP and Iba1 expression and inhibit proinflammatory cytokines IL-1β and TNF and enhance anti-inflammatory IL-10 (144). Interestingly, conditioned medium from CGRP (calcitonin gene-related peptide)-primed DPSCs expressed high levels of inflammatory mediators including CXCL1 and CXCL8, both are ligands of CXCR2 (151). The CXCL1-CXCR2 signaling axis contributed to BMSC-induced descending pain inhibition in the masseter muscle TL model (8).

Exosomes are a class of small extracellular vesicles (sEVs) secreted by all types of cells. Although there are distinct markers for different classes of sEVs, such as CD61/CD63 for exosomes and TyA/C1q for ectosomes (152), currently, there is no practical way to obtain a pure exosome preparation without contamination by other sEVs such as ectosomes (microparticles) that are derived from the cell membrane. Nevertheless, ectosomes may contain similar cargo and present an analogous effect. The use of exosomes may avoid pulmonary embolism seen in stem cell therapy (153). While exosomes from MSCs are a promising alternative for MSCs, the antihyperalgesic effect of exosomes lasts much shorter than their parent cells, likely due to their weak immunomodulatory effect compared to their parent MSCs (150).

Collectively, MSCs produce pain relief through their interactions with the host immune system (Figure 2). The interactions between MSCs and host immune cells involve autocrine signaling of MSCs and the secretion of their secretome/exosomes, as well as inflammatory mediators and their receptors. These interactions lead to the promotion of anti-inflammatory phenotype, suppression of glial hyperactivity, and activation of endogenous opioids, resulting in pain relief. The CXCL1-CXCR2 signaling pathway has been suggested to play a role in enhanced MOR expression and pain relief, although *in vivo* interactions between chemokines and opioid receptors during MSC intervention after injury require further investigation. Multiple other mechanisms may also underlie the pain-relieving effect of MSCs. One such mechanism is the neuropeptide galanin, which contributes to BMSC's antihyperalgesia by suppressing protein kinase M*ζ*, a brain-specific protein kinase C isoform with persistent activity, and its receptor GalR1 (154, 155). Improvement of oxygen supply has also been correlated with bone-marrow cell-induced pain relief in patients with limb ischemia pain (156), and reduced reactive oxygen species have been linked to antinociception in rats with spinal nerve ligation (157). Hence, the analgesic efficacy of MSCs and their secretome exhibits immense potential, and the underlying mechanisms responsible for alleviating chronic pain in diverse preclinical pain models, such as temporomandibular joint-osteoarthritis (TMJ-OA), orofacial myalgia, and trigeminal nerve injury, warrant further investigation.

## Clinical applications of stem cells to treat orofacial pain

**Figure 3:**
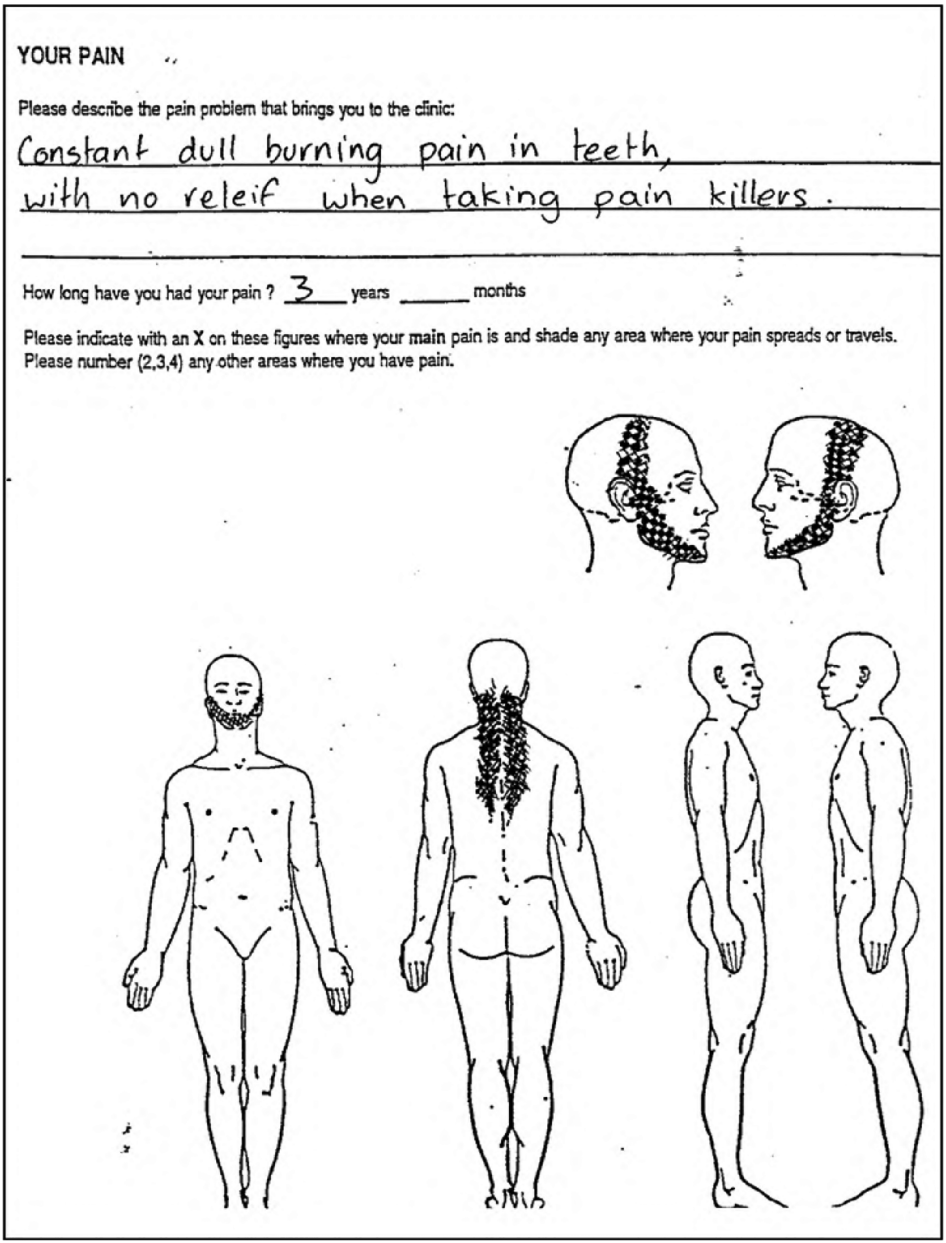
Classical description and pain map of neuropathic pain, then causing a secondary temporomandibular disorder. *neuropathic pain is “constant” and “burning”. *Neuropathic pain has a poor response to opioid drugs such as codeine. *Development of secondary TMD in the masseter, temporalis, occipital and supraspinal muscle groups.

Chronic pain is a specific symptom of several orofacial disease states, including trigeminal neuropathy, pain from oral mucositis, and TMJ disease. However, a confounding variable in assessing stem cell outcomes to treat orofacial pain is the presence of multiple concurrent diseases with different pain states (e.g., classical tissue vs. neuroinflammation-related pain) and how stem cells may prioritize tissue regeneration. For example, to what tissue will stem cells migrate in a patient with trigeminal neuropathic pain, associated myofascial pain, and an acute dental infection? To obtain accurate research outcome data, the dental researcher must, in turn, identify and list the multiple orofacial pain states prior to stem cell treatment (Figure 3) (158). In this subsection, we will provide a detailed overview of the clinical concepts and guidelines for utilizing stem cell therapy in the treatment of orofacial pain. We will outline the specific steps and procedures that must be followed to ensure safe and effective use of this therapy in the clinical setting.

### Patient selection and exclusions

Autologous MSCs have a wide range of potential therapeutic applications for medical conditions such as arthritis and multiple sclerosis (159, 160). Dental applications of stem cells could be particularly advantageous in older adults as the incidence of orofacial pain in patients aged 46 and older is higher than other age groups (161). Burning mouth syndrome was the most prevelant orofacial pain condition in older adults followed by TMD, trigeminal neuralgia, sinusitis and odontogenic referred pain (161). Moreover, several non-analgesic drug classes such as benzodiazepines, anti-depressants, sleeping aids among others are used as treatment modalities due to the chronic nature of the pain (161). Nevertheless, in dental practice, there are several patient exclusions for stem cell therapy:

1. Active cancer due to the risk of acquiring cancer cells and injecting these cells into healthy tissue that can inadvertently spread the cancer. The dentist would require a clearance letter from the medical doctor or oncologist stating the cancer is under management and stem cell treatment can proceed.
2. Systemic and local infections of the face and oral mucosa (e.g. acute necrotizing ulcerative gingivitis) would preclude harvesting from local bone and adipose tissue.
3. Drugs that may affect surgery such as high dose anticoagulants, or potentially affect stem cell viability and cell communication such as methotrexate, immunomodulatory drugs and steroids.
4. Psychiatric patients who are noncompliant with medication.
5. Poor oral hygiene.
6. Pregnancy and lactation.

### Selection, harvesting and processing of stem cells for clinical use

The choice of stem cell type for therapy depends on several factors because each source has its advantages and disadvantages (see Table 1). Adipose-derived stem cells (ADSCs) are versatile cells that can differentiate into various cell types, such as endothelial cells, neurons, chondroblasts, and osteoblasts that subsequently form blood vessels, nerves, cartilage, and bone. On the other hand, hematopoietic-derived stem cells (HDSCs) are also multipotent but lack adipogenesis differentiation. Both ADPSCs and HDSCs have a very low intrinsic risk of complications when administered. Complications can occur but are preventable as they typically result from operator error when performing surgical harvesting from the donor tissue or subsequent incorrect injection of stem cells. Allogeneic stem cells from healthy donors and umbilical cord stem cells (UCSCs) are readily available from laboratory sources but have the potential to induce graft vs. host disease (162). Autologous induced pluripotent stem cells have great potential but show increased tumorigenicity and have a low yield in the conversion from somatic cells to stem cells, and there are high laboratory costs involved. Similarly, embryonic stem cells can form almost all types of tissue cells but demonstrate tumorigenicity and have ethical constraints (163). Collectively, while several sources of stem cells exist, cellular differentiation as well as safety profile make a select few sources the preferred choice for clinical use.

**Table 1:** Sources and applications of stem cells for dentistry and orofacial pain; advantages and disadvantages.

Cell source	Dental application	Advantages	Disadvantages
Autologous abdominal ADSCs (and lateral thigh & pectoral fat)	Neuropathic pain Periodontal bone loss Bone augmentation for implants TMJ problems Salivary gland Paresthesia Facial atrophy	Safe No systemic side effects Minimal surgical risk Repeatable	Requires general surgeon Increased cost Discomfort and bruising at donor site
Autologous orofacial ADSCs (buccal fat, submental fat)	Neuropathic pain Periodontal bone loss Bone augmentation for implants TMJ problems Salivary gland Paresthesia Facial atrophy	Safe Adipose acquisition can be performed by oral surgeon	Higher risk of marginal mandibular nerve injury from submental fat Microbial contamination from intraoral acquisition of fat
Autologous bone marrow & blood HDSCs	TMJ problems Periodontal bone defects Bone augmentation for implants	Intraoral bone marrow acquisition possible Simple intravenous blood acquisition Repeatable Cell banking possible	Low cell numbers from blood Needs cell culture proliferation Increased cost
Autologous IPSCs	All dental tissues	minimal surgery to acquire somatic cells Cell banking Repeatable treatments	Low yield of conversion from somatic to pluripotent state Higher risk of tumorigenicity High laboratory costs
Allogeneic stem cells (ADSCs, HDSCs, IPSCs)	All dental tissues for IPSCs	nil surgery Repeatable “On demand” cells	possible graft versus host disease Autoimmune disease Chimera DNA medicolegal problems
Umbilical cord blood SCs	All dental tissues	Nil surgery Repeatable “On demand” cells Low risk of autoimmune issue	limited cell numbers from cell expansion in culture Chimera DNA medicolegal problems
Embryonic SCs	All dental tissues	nil surgery Repeatable “on demand” cells unlimited cell numbers Cell banking	Higher risk of tumorigenicity High laboratory costs Chimera DNA medicolegal problems Ethical constraints
Chemotactic peptide derived SCs	Potentially for all dental tissues	No surgery or blood collection needed Repeatable injections to site Peptides can be synthesized, standardized and validated by mass spectrometry	experimental—no clinical studies Unknown risks but likely low risk as chemotactic peptides are endogenous to humans

ADSCs, adipose derived stem cells; HDSCs, hematopoietic derived stem cells; IPSCs, induced pluripotent stem cells; USCs, umbilical cord blood stem cells; ESCs, embryonic stem cells; CPSCs, chemotactic peptide stem cells; TMJ, temporomandibular joint.

ADSCs acquired from abdominal fat or HDSCs from bone are also the preferred choice of cells for the management of orofacial pain when ensuring safety and compliance with established laboratory protocols for harvesting. The harvesting of adipose tissue from the abdomen is a minor surgery that carries a low risk of injury to important structures. The surgical process involves draping and topical disinfectants, which result in minimal bacterial counts in the donor adipose tissue. While harvesting submental or buccal fat may be possible, it is important to consider the location of the marginal mandibular nerve to avoid injury. Harvesting from the buccal pad of fat requires an incision through the oral mucosa, which can result in significantly higher bacterial counts, including pathogenic species, in the donor fat and, thus, in the stem cell injection. Narrow gauge liposuction cannulas may cause local bruising and discomfort postoperatively. Alternatively, bone marrow from the iliac crest or mandible may be considered as a source of cells. However, similar advantages and disadvantages are present, particularly regarding bacterial contamination during processing and the final injection of cells. One significant advantage of ADSCs over HDSCs is that ADSCs have a five-fold lower stem cell senescence rate, indicating their greater proliferative potential and resultant higher cell numbers (33).

### Cell numbers and phenotype

**Figure 4:**
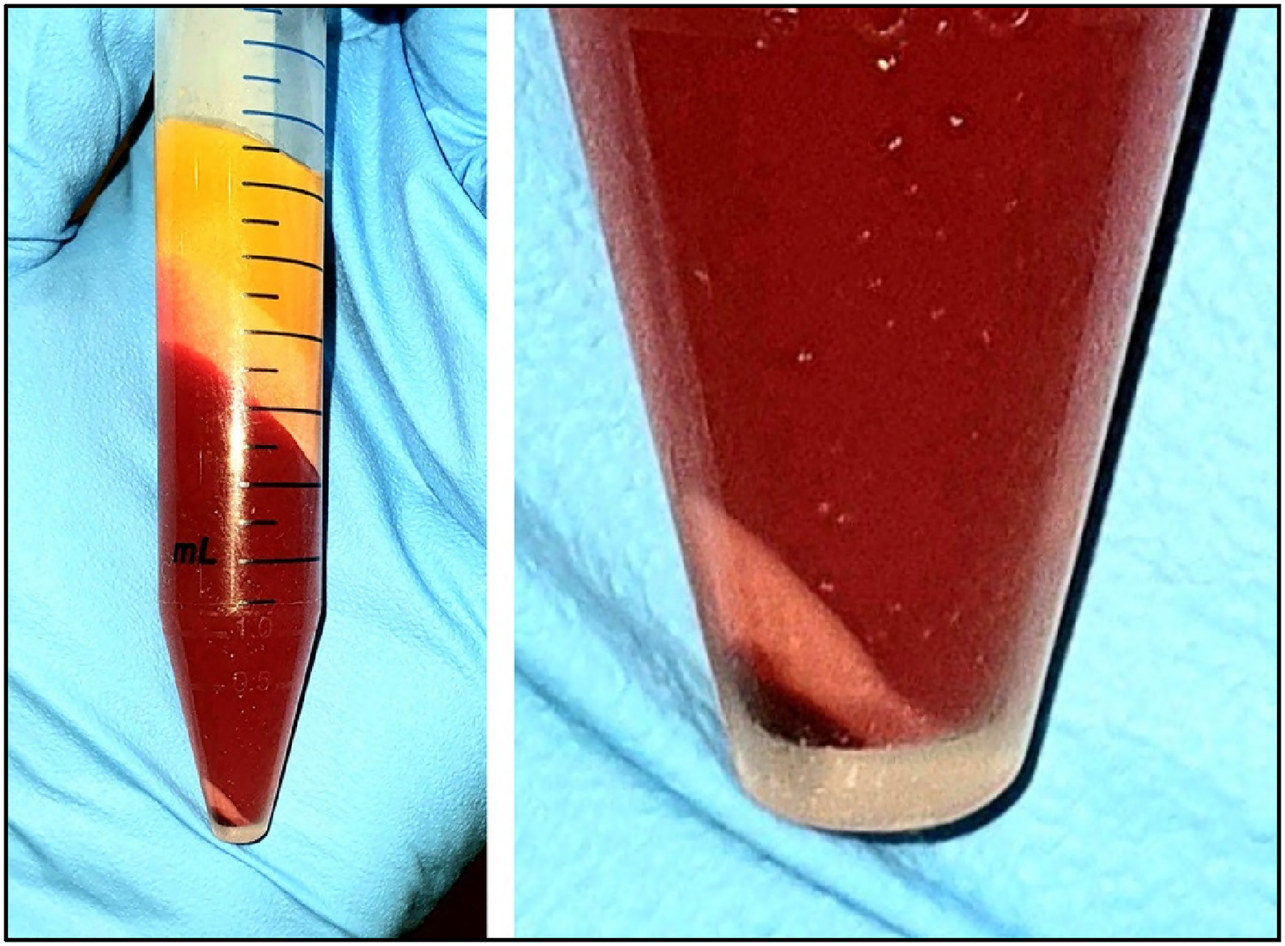
Image of the lipoaspirate after mechanical emulsion and centrifugation. Upper layers of yellow adipose fat and free lipid. Middle liquid phase layer of saline, plasma, local anesthetic, red cells. Lower layers of ADSCs (pinkish white) and small dark red cell layer at the very bottom.

The International Myeloma Foundation (IMO) guidelines suggest that stem cell transplants for treatment of myeloma have a minimum requirement of two million cells per kg of body weight (164). However, the number of stem cells needed to achieve a therapeutic effect for orofacial pain relief has not been established due to the wide spectrum of heterogeneity of the disease state (for example, TMJ arthritis and facial neuropathy) when measuring variables such as pain intensity, duration of the disease and presence of multiple pain sites. Medical studies often report the acquisition of 200–300 gm of abdominal fat obtained by tumescent liposuction to yield 50–60 million cells from the collagenase enzymic digest method (0.3 million cells/gm). This cell number was used on a case series using autologous ADSCs to treat trigeminal neuropathic pain with reported safety and preliminary efficacy (165). Recent improvements in laboratory protocols harvesting lower amounts of adipose tissue (1–5 gm) combined with a non-enzymic mechanical emulsion and centrifugation approach has yielded higher cell viability and cells/gm. The 2014 study reported cell viability at 75% vs. the current non-enzymic method with viability >95% (Figure 4). Despite the smaller amount of abdominal fat collected in the latter method, the yield of ADSCs may be considered sufficient to treat localized sites such as the TMJ, periodontal ligament and trigeminal branch neurovascular pain if using the IMO guide.

**Figure 5:**
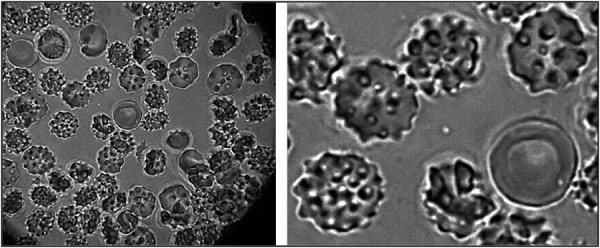
ADSCs freshly acquired from the stromal vascular fraction (SVF). ADSCs show excellent development and activation. Centrally located red cell in the image shows size comparison of the cells. Image taken at 1000X magnification with an oil immersion objective.

**Figure 6:**
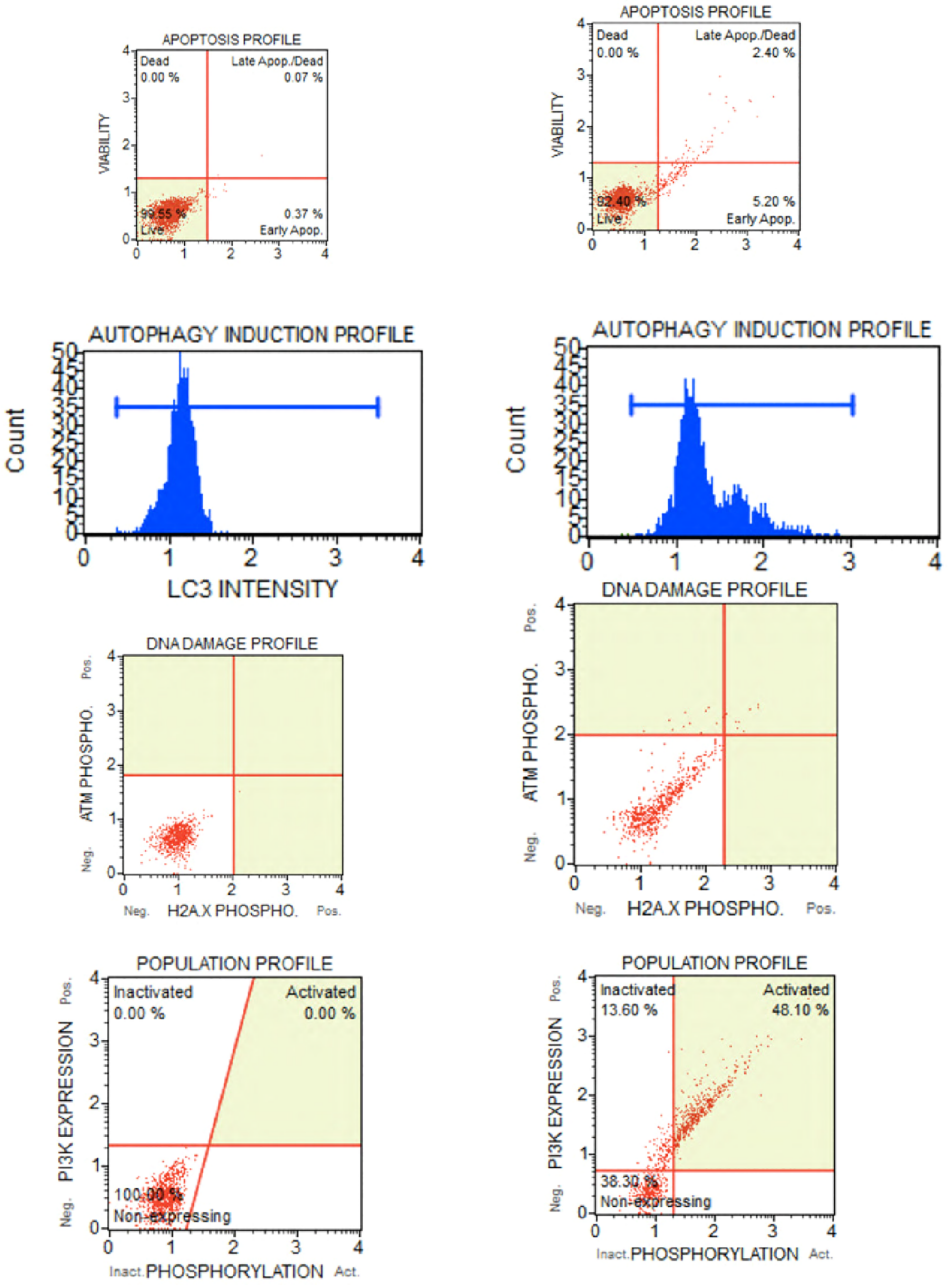
Samples of flow cytometer assays from patients undergoing stem cell therapy for orofacial pain. A small aliquot of ADSCs are used for quality control assessment of the adipose tissue processing. Assays conducted with a MUSE Cell Analyser (Merck Millipore Ltd.) and performed for annexin V (apopotosis), autophagy (cell breakdown), H2A.X (DNA damage) and tumorgenicity marker Pi3. (**A**) assays are baseline measurements taken 10 min after stem cell administration, (**B**) ADSCs subsequently exposed to high dose gamma radiation for several days to simulate stem cell deterioration and changes to the cell profiles that occurs with aging, certain disease states and medical radiotherapy.

**Figure 7:**
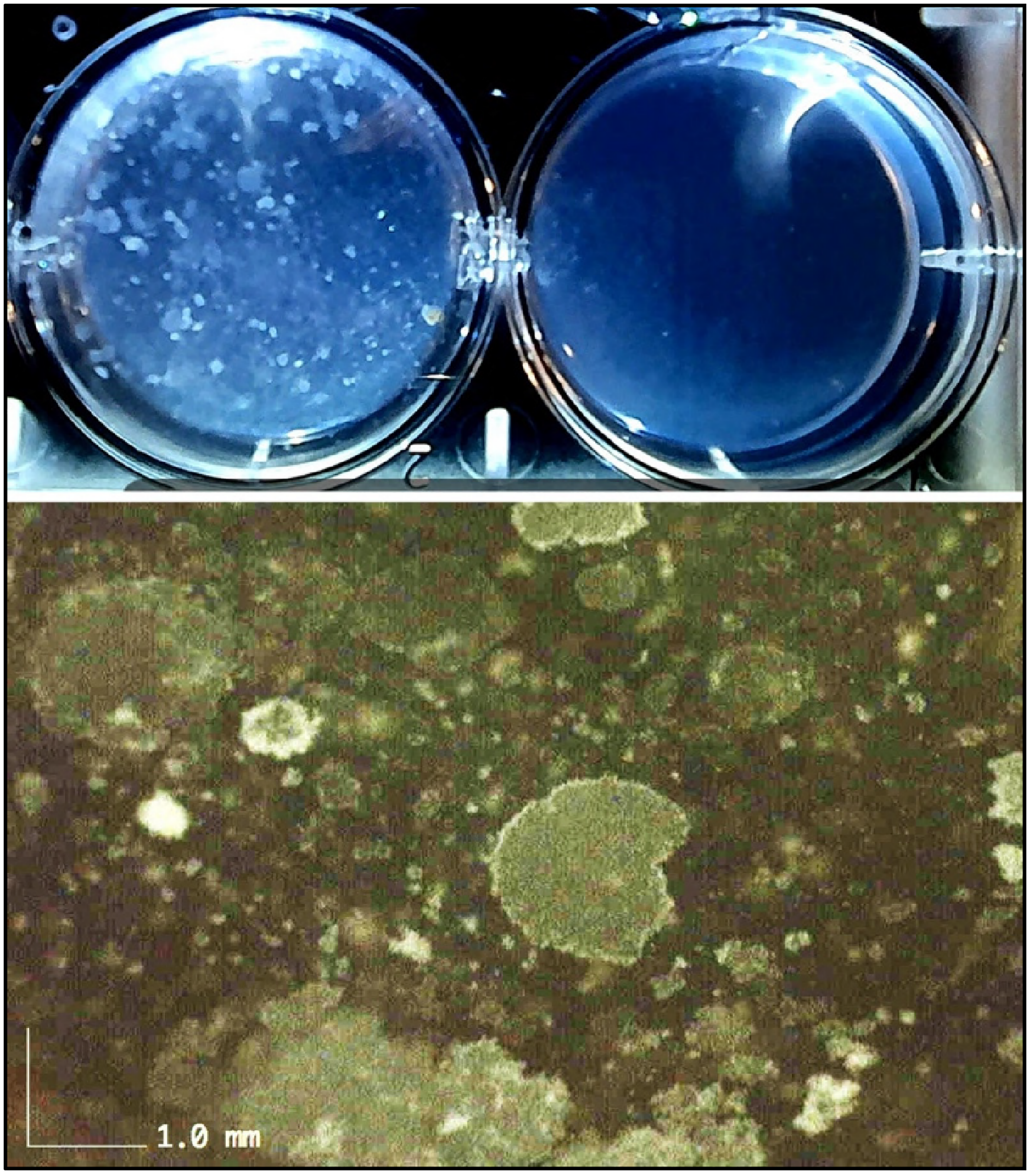
Confirmation of proliferative aspects of ADSCs. Macrosopic upper image taken at 4 days after ADSCs plated in wells with 8 ml hydrogel. Left well shows cell proliferation throughout the gel matrix and right well used as control gel. Higher resolution lower image at 50X shows multiple colony forming units (CFUs) indicating excellent stem cell proliferative characteristics.

Cellular phenotype and cell health are critical for the clinical outcome. Direct brightfield microscopy at a high magnification of 1000X with an oil immersion objective is a simple and rapidly accomplished procedure in a dental clinic setting to demonstrate real-time activation of stem cells from adipose tissue (Figure 5). Cell identity and cell health is achieved by flow cytometry CD markers. The International Society for Cellular Therapy (ISCT) has designated several markers positive for CD73, CD90, and CD105 and negative for CD14, CD19, CD34, and CD45 that define and confirm ADSC phenotype. Flow cytometry can further investigate useful cell health parameters of apoptosis, autophagy, DNA damage, and the presence of cancer markers such as Pi3 (Figure 6) (166). Annexin V is a marker for apoptosis (programmed cell death) and detects phosphatidylserine (PS) translocation to the external environment. Autophagy is involved in the stem cell phases of activation and differentiation. It plays a dual role in cancer as a tumor suppressor and promoter. H2A.X (DNA damage) is increased during the promotion of self-renewal for stem cells. It is a sensitive marker to examine DNA damage and subsequent repair. Pi3K (phosphoinositide 3-kinase) promotes hematopoietic stem cell activation and plays a crucial role in mitogenesis, proliferation, prevention of apoptosis and maintenance of multipotency in mesenchymal stem cells. It is overactivated in certain cancers. One additional step in the laboratory protocol is to assess the proliferative potential of the stem cells. A small aliquot of the ADSCs can be plated in a hydrogel to visually examine the amorphous cell proliferation and the ability to form functional colony-forming units (CFUs) (Figure 7).

### Administration of stem cells for orofacial pain

**Figure 8:**
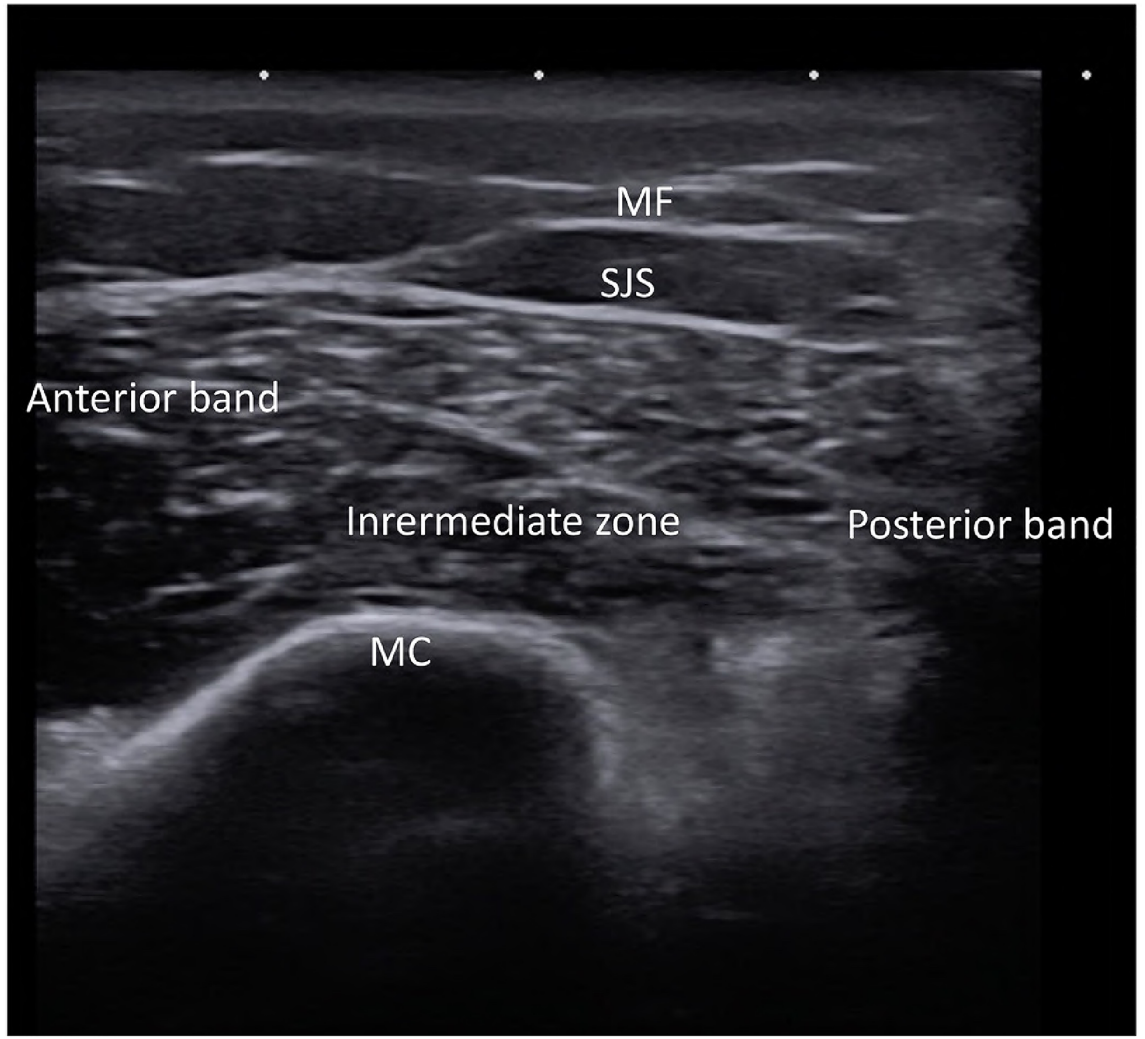
Ultrasound of right temporomandibular joint of healthy subject showing normal architecture of mandibular condyle (MC), mandibular fossa (MF), superior joint space (SJS) and the three sections of the meniscus (anterior, intermediate and posterior).

Stem cell lipoaspirate (SVF) is commonly administered directly into the target site for periodontal regeneration, bone augmentation, TMJ arthritis and salivary gland regeneration. Therefore, this approach appears to be the preferred procedure for cell delivery for orofacial pain conditions. Moreover, no studies have been conducted to assess the outcomes from IV infusions to treat orofacial pain. After the application of a topical anesthetic, an injection of the topical anesthetic with a narrow 27–30 gauge needle into the target site is accomplished. This prevents significant pain that can occur with the larger bore 21–23 gauge needle containing the stem cells. The larger gauge is needed to prevent microfat needle blockage and any potential damage to cells from shearing actions on cell membranes in narrow bore needles that would decrease viability. Injections of the stem cell lipoaspirate (termed the stromal vascular fraction, SVF) are directed to the pain sites. Perineural injections are placed into the region of the major maxillary and mandibular trigeminal nerve branches to treat widespread neuropathic orofacial pain. Importantly, the injections are not injected into the nerve directly as neuroma formation can develop from needle bevel trauma. Ultrasound (US) guided needle placement is required for exact needle insertion into the superior joint space of the TMJ (Figure 8).

**Figure 9:**
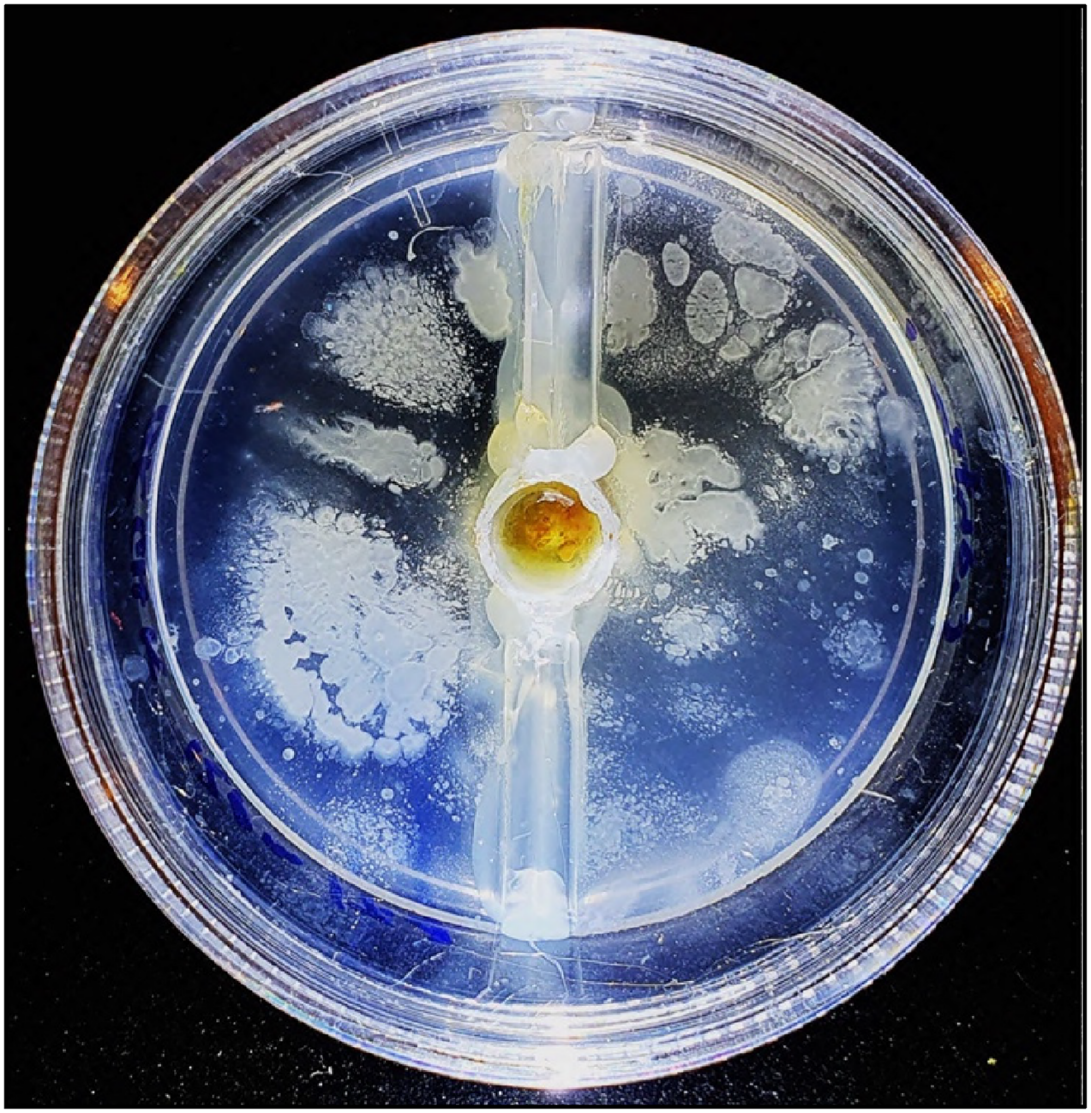
Peptidic chemotactic alginate-based hydrogel. Drop of blood placed in central well with perforations in the central well to allow cell migration into the surrounding peptide hydrogel. Image taken at 4 days incubation at 37C and show excellent hematopoeitc stem cells (HDSCs) migration and proliferation.

A vehicle for stem cells as well as a suitable environment for stem cell growth and differentiation, is provided by scaffolding materials such as hydrogels. Hydrogels are three-dimensional networks of hydrophilic polymers that can absorb and retain large amounts of water, making them an attractive option for tissue engineering applications. Emerging medical evidence suggests that injectable sodium alginate biodegradable hydrogels are beneficial for cardiac regeneration and joint arthritis (167, 168). Sodium alginate is widely used by dentists and is currently undergoing *in vitro* research as it can incorporate live stem cells (169), while also possessing several other advantages, such as biocompatibility, low manufacturing cost, and cross-linkages with calcium and magnesium ions for improved mechanistic parameters. The existing knowledge base of alginate physicochemical attributes and ease of administration by dentists also make it an attractive option. Moreover, the hydrogels can incorporate various cofactors, such as peptides, polyphenols, antibiotics, and vitamins, which can promote cell expansion and direct cell fate toward the target tissue. However, it is crucial to scrutinize oral hydrogels *in vivo* due to the potential contamination and expansion of oral microbes and fungal elements within the biogel. Recent advancements in hydrogels have demonstrated the use of stem cell chemotactic peptides, allowing stem cells from the local tissue niche, circulating HDSCs, and ADSCs to migrate directly to the hydrogel. This new development eliminates the need for donor surgery and only requires a series of local anesthetic injections, the hydrogel placement, and the crosslinking agent. This approach permits simple, low-cost, and gradual increments in tissue and is ideal for certain medically compromised patient groups (Figure 9) (170). Therefore, a direct administration of hydrogel-loaded stem cells is delivered to the target site for optimum cell number and cell-favorable local environment.

### Training and dentolegal issues

**Figure 10:**
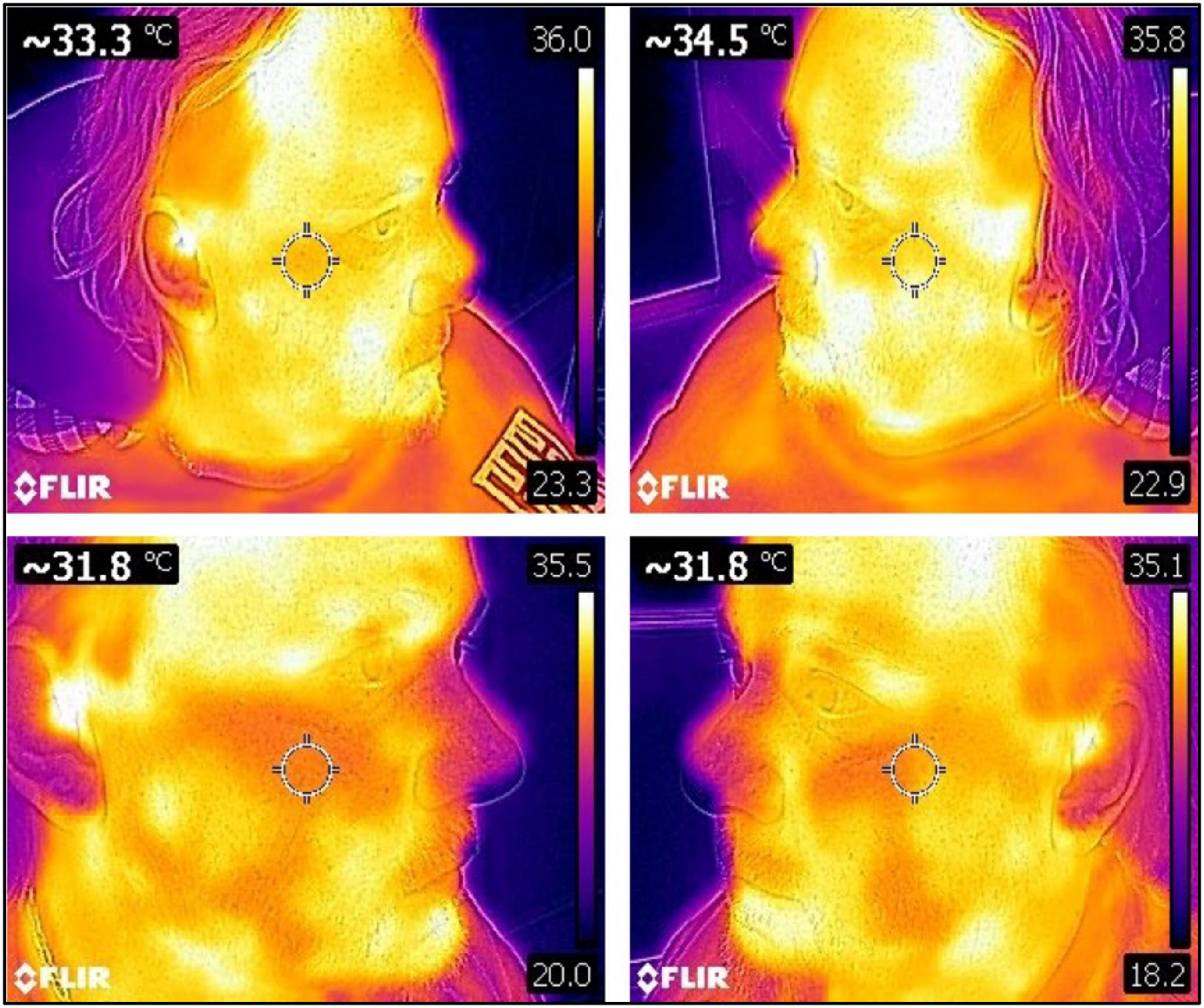
A 45 year old male with facial neuropathic pain and sympathetically maintained pain. There was six year pain history of right maxillary and mandibular trigeminal neuralgia. Previous treatments included seven neurosurgical operations (microvascular decompression, pulsed radiofrequency, nerve ablation), multiple Botox and ketamine/lidocaine infusions, high dose carbamazepine and gabapentin. Baseline thermograms before stem cells showed sympathetically maintained pain in the right maxilla with surface temperature differential −1.2C on the painful right maxilla (33.3C) compared to the left non-painful maxilla (34.5C) (normal range +/- 0.2C). Thermograms repeated at five weeks after stem cell injections showed nil surface temperature differential (bilateral 31.8C) showing resolution of neurovascular pain.

Diagnosing orofacial pain can be challenging due to the intricate anatomy of the head and neck region, which involves exocrine glands, neurovascular components, and the musculoskeletal system. Pain typically results from tissue injury, triggering a cascade of peripheral nociception, retrograde transmission, and central processing. As a multidimensional biopsychosocial phenomenon in humans, orofacial pain involves various factors contributing to its development and persistence. Patient safety mandates that clinicians have adequate clinical and laboratory training in the use of stem cells to treat orofacial pain and for the regeneration of dental tissues such as the periodontium (171). The preoperative planning phase must assess any significant psychological issues such as anxiety, depression, anger, and frustration, particularly where there has been a long history of multiple clinicians, misdiagnoses, and costly repetitive failed treatments. A critical aspect of achieving patient satisfaction and postoperative compliance with stem cell therapy is identifying and explaining to patients what realistic vs. unrealistic expectations from the treatment (172). Ongoing research will identify beneficial outcomes from treatment for orofacial disease and symptoms. The trigeminal region is localized and should add knowledge to discriminate subsets of stem cell reparative mechanisms involving paracrine anti-inflammatory effects, expression profiles (secretome), intercellular peptide communication, and differentiation characteristics of the tissue. Outcomes will rely on qualitative information with validated questionnaires such as the visual analog scale (VAS) and the McGill Pain Questionnaire (MPQ). Incorporating additional quantitative objective measurements can be valuable in diagnosing orofacial pain. For example, thermography can be used as a non-invasive tool for detecting neurovascular disturbance, while von Frey neurosensory testing, which measures mechanical allodynia and hyperalgesia, can provide reassurance to patients that their “invisible pain” is present, real, and improving (Figure 10). Stem cells play a crucial role in the healing of various dental injuries, including tooth extraction sockets, soft tissue incisions, oral mucosal microlesions, and traumatic ulcers caused by mastication. By boosting cell numbers during the healing phase after an injury, stem cell therapy can promote dental regeneration and help alleviate orofacial pain.

## Conclusions

Biological treatments such as autologous cellular therapies are a major advancement in 21st-century medicine, especially for patients with chronic orofacial pain. The use of biotechnology, such as stem cell intervention, has traditionally been viewed conservatively by national health authorities, with authorization for dentists being lacking on an international level. Regenerative medicine therapies have not been approved by the FDA nor EMA for the treatment of any orthopedic condition, such as osteoarthritis, tendonitis, disc disease, tennis elbow, back pain, hip pain, knee pain, neck pain, or shoulder pain or orofacial pain. However, the Australian Government approved the use of autologous stem cells by dentists in 2018 after assessing their safety and potential efficacy in dental disease, and orofacial pain states (173). In fact, dentists have a unique advantage in accessing additional HDSCs and ADSCs from readily available sources, such as the maxilla, mandible, and local fat deposits, including the buccal pad of fat and submental chin fat. Traditional drug treatments like first-line anti-neuropathic drugs and second-line opioids often come with problematic side effects and poor patient compliance. Autologous stem cells, on the other hand, offer significant benefits over drug treatments by avoiding side effects and improving patient compliance. These cells can deliver multiple therapeutic effects, such as anti-inflammatory actions, immune system modulation, and regeneration of damaged tissue to a functional state that collectively can aid in the resolution of pain states.
